# Fertility and endocrinopathies among adults with β-thalassemia major treated at Dubai thalassemia center

**DOI:** 10.3389/fendo.2026.1746560

**Published:** 2026-07-02

**Authors:** Rabah Almahmoud, Amal Hussein, Fatheya Al Khaja, Ahmed Farrag Soliman, Hany Dewedar, Sarah Mathai

**Affiliations:** 1 Department of Clinical Sciences, College of Medicine, University of Sharjah, Sharjah, United Arab Emirates; 2 Department of Family & Community Medicine and Behavioral Sciences, College of Medicine, University of Sharjah, Sharjah, United Arab Emirates; 3 Dubai Thalassemia Centre, Dubai Health Authority, Dubai, United Arab Emirates; 4 Department of Pediatrics, Christian Medical College, Vellore, India

**Keywords:** deferariox, deferiprone, endocrinopathies, infertility, oral chelators, thalassemia major

## Abstract

**Introduction:**

β-thalassemia major (BTM) is made up of group of quantitative anemias characterized by severe anemia and hemochromatosis from frequent blood transfusion. Iron overload in the endocrine system (pituitary gland, thyroid and parathyroid glands, pancreas, and gonads) resulting in different endocrinopathies is a major challenge among BTM patients especially among adults. This study aims at describing different endocrinopathies; namely short stature, hypothyroidism and diabetes mellitus among adults treated at Dubai Thalassemia Center in United Arab Emirates.

**Materials and methods:**

This study is observational retrospective cohort in nature, of all adult patients with BTM aged above 18 years who attended the Dubai Thalassemia Centre during the period of November 2019 and May 2021, were extracted from patients’ electronic medical records.

**Results:**

A total of 200 adults with BTM above 18 years of age were enrolled in this study. Females constituted 54.5% (n=109) of the sample while 45.5% (n=91) were males. The annual average pre-transfusion hemoglobin level was 9 gm/dL in 81.5%, n=163, while it was low (<9 gm/dL) in 18.0% (n=36) patients. The mean height of all male patients was 165.4 cm (SD = 6.05) and for females it was 154.11 cm (SD = 5.67), while the mean weights of all male and female patients were 60.4 kg (SD + 12.74) and 55.8 kg (SD = 14.60), respectively. Of all female patients, the mean age of menarche was 15 years. The prevalence of Hypogonadism is 23%, Diabetes Mellitus 15%, Hypothyroidism 11%, Hypoparathyroidism 9% and Short Stature 1.5%.

**Conclusion:**

Despite the improved care and introduction of oral chelators, the prevalence of endocrinopathies namely hypogonadism, short stature, hypothyroidism, hypoparathyroidism and diabetes mellitus still pose a major concern for patients with BTM. More emphasis needs to be placed on improving compliance with chelation, and further studies are required to investigate new therapies options to reduce these complications.

## Introduction

β-Thalassemia major (BTM) is made up of a group of quantitative anemias that is inherited as an autosomal recessive condition were either there is reduced production of the β-globin gene (β+) or complete absence of production (β0) located on chromosome 11. Patients with BTM have excess α-chains which in turn lead to ineffective erythropoiesis and shorten red blood cells’ life span due to reduced quantity of β-globin chains, hence reducing the adult hemoglobin (HbA) (1, 2). So far, there has been over 200 point mutation that were identified. BTM presents soon after birth at around six months of life when fetal hemoglobin production is switched off and adult hemoglobin is synthesized. Infants often present with failure to thrive, severe pallor, hepatosplenomegaly due to the extramedullary hematopoiesis. Historically, BTM has been common in the Mediterranean region, Middle East, and Southeast Asia. However, due to immigration, the condition is increasingly seen in North America and Western Europe as well (3). The estimated incidence world wide of BMT is 68,000, while the prevalence is 1.5% globally (2). The anemia, which is hemolytic if left untreated or poorly treated, is associated with a distinct facial appearance (2, 4, 5). Nearly one century ago, Dr. Thomas Cooley first described these children as having frontal bossing, malar eminence, jaundice, and splenomegaly (6). The disease was named after him soon after. The diagnosis of BTM is based on clinical suspicion in infants with these findings in addition on hypochromic microcytic anemia on complete blood count and complete absence of the adult hemoglobin on high-performance liquid chromatography or hemoglobin electrophoresis. Left untreated, the natural history of BTM would be death from chronic anemia and cardiac decompensation. Early and regular blood transfusion with the aim to hyper-transfuse and suppress the patient’s own ineffective erythroid production has saved these patients from death in the first decade of life (7). However, these transfusions are complicated by iron deposition in different organs including the heart, liver, and endocrine system (pituitary gland, thyroid and parathyroid glands, pancreas, and gonads). The endocrine system seems to be sensitive to unbound iron, for example, the thyroid gland; and other endocrine organs; are susceptible to cellular damage, and cell death caused by the reactive oxygen species (ROS) generated by the unbound iron which is released from chronic hemolysis and is worsened by regular blood transfusion (8). ROS causes severe oxidative damage through lipid and protein present in the cell membranes which causes ferroptotic cell death (9, 10). There is emerging data that mitochondrial dysfunction from accumulation of α chains in the cells causes increased demands on mitochondria for energy production for erythropoiesis and clearance stress and ultimately mitochondrial and cell destruction. Enzymes like pyruvate kinase reduces the formation of ROS and oxidative stress (11–14). Another enzyme called Unc-51-like kinase 1 (*Ulk1*) promotes cells autophagy of excess α chains, a mutation in genes encoding for this enzyme have been shown to increase α chain clearance (15, 16).Currently used oral iron chelators has been show to attenuate the oxidative damage and mitochondrial stress (17).

Endocrinopathies among BTM patients is well documented in the medical literature. There is direct evidence that the chelation status plays a major role in the pathogenesis. Data shows that poorly chelated patients have shown six times higher risk of developing hypothyroidism compared to well chelated patients with a prevalence of hypothyroidism ranging from 12 to 20% (18, 19).

Impaired growth is one of the most common endocrinological complications that patients with BTM have with nearly half of patients being affected (20).

Hypogonadism is regarded as the most common endocrine abnormality in several studies around the world (21, 22). There is evidence that patients with BTM fail to enter puberty if left without chelation or if ferritin is extremely high, that is above 9000 µg/L, where the damage is irreversible (23, 24). The gonads are highly sensitive to iron, and low testosterone has been negatively correlated with pituitary iron (25). Male hypogonadism can be primarily due to testicular failure in which testosterone level is low while FSH and LH hormones are high or secondary due to hypothalamic-pituitary axis failure in which testosterone level as well as FSH and LH hormones are low (26). A study from the United Arab Emirates (UAE) found that patients with ferritin levels above 2500 µg/L had almost three times higher chances of developing hypogonadism compared to patients with ferritin levels below 1000 µg/L (27). However, recent data suggest that, even with good chelation (ferritin level below 1000µg/L), neither glucose dysregulation nor hypogonadism could be prevented (28).

Poor compliance to iron chelation therapy poses a huge risk despite the introduction of oral chelators namely Deferiprone and Deferasirox. Non-compliance rate was reported in a Malaysian study to be 24.7% among patients older than eight years of age (29). The non-compliance was observed among all the three iron chelators was shown in a metanalysis were it ranged from 3.9-29.4% among patients on Deferoxamine, 5.1-17.6% among Deferiprone and 1-14.7% among BTM patients on Deferasirox (30).

The estimated prevalence of β thalassemia carrier is 2.3% (31). This relatively high prevalence is explained by the high rate of high rate of consanguineous marriages reaching as high as 50.5% (32).

The emirate of Dubai, which is one of the seven emirates of the UAE, has the Dubai Thalassemia Center that serves patients with BTM and other hemoglobinopathies from all parts of the country. Two oral iron Chelators are used in the Centre: Firstly, Deferiprone which was introduced in 2000. Secondly, Deferasirox which was introduced in 2007.

This study aims to describe the prevalence of endocrinopathies, namely diabetes mellitus, hypothyroidism, hypoparathyroidism, hypogonadism, and fertility, among adults with BTM in the UAE in the post oral iron chelators era.

## Materials and methods

### Study design

This study is an observational retrospective cohort in nature, where retrospective data, of all adult patients with BTM aged above 18 years who attended the Dubai Thalassemia Centre during the period of November 2019 and May 2021, were extracted from patients’ electronic medical records.

### Collection of data

In this retrospective study, data were extracted from the medical records related to patients’ sociodemographic and clinical characteristics. Two separate data collection sheets were used to extract data from the records of male and female patients. For both genders, sociodemographic and physiological characteristics included date of birth, gender, nationality, patient’s weight, and height. Extracted clinical data included full blood count, fasting plasma glucose level, thyroid profile, bone health, and liver function tests. The diagnosis of BTM was initially done based on the clinical presentation and was then confirmed by DNA analysis and hemoglobin electrophoresis. In addition to data extraction, patients were asked to fill out a questionnaire to collect additional data related to their diet, age of puberty, marital status, and number of children. All adults with BTM who attended Dubai Thalassemia Centre were asked for written consent to be enrolled in the study. Patients who either refused to be enrolled or had Bone Marrow Transplant were excluded from the study.

### Data analysis

Data were initially entered into Microsoft Excel and were then exported to the Statistical Package for Social Sciences (SPSS) program, version 28.0, for data coding and analysis. Descriptive data analysis was conducted using frequency distribution statistics (counts and percentages) for qualitative variables and measures of central tendency (means and medians) and variability (standard deviation and interquartile range) for quantitative data. Statistical tests were conducted as appropriate to the type of analyzed data. Means and standard deviations (SD) were reported for normally distributed data while medians and interquartile range (Q3-Q1) were reported for skewed data. To test the normality of quantitative data, Kolmogorov-Smirnov and Shapiro-Wilk tests were used. The Chi-square test was used to study the association between two categorical variables. Mann-Whitney U test was used to test the equality of two medians, while the Wilcoxon Signed Ranks test was used to compare a median against a reference value. Missing data were handled using the pairwise deletion approach. A p-value below 0.05 was considered statistically significant. When multiple correlations were performed, statistical significance was corrected using Bonferroni’s correction. Therefore, when 15 correlations were performed, a corrected significance threshold of p < 0.003 was used (alpha=0.05/15 = 0.003).

To measure the prevalence of diabetes mellitus, hypothyroidism, hypogonadism, hypoparathyroidism, hypogonadism and short stature, existing cases were identified through searching for each diagnosis in the problems list extracted for each patient from the thalassemia center database. Fasting plasma glucose (FPG) level below 100 mg/dL was considered normal while FPG ≥126 mg/dL indicated diabetes (25, 26). Anemia was diagnosed based on hemoglobin level below 9 g/dL (33–35), Thyroid-Stimulating Hormone (TSH) was dichotomized into two groups: normal (TSH between 0.3 and 4.2 ulU/ml) and elevated (TSH > 4.2ulU/ml). Hypoparathyroidism: low serum levels of calcium, elevated phosphorus and inappropriately low or low PTH levels. Male hypogonadism is defined as failure of the testes to produce sufficient testosterone, sperm, or both. It is characterized by low serum testosterone levels (< 280 ng/dl) and can be caused by testicular failure (primary) or hypothalamic-pituitary dysfunction (secondary). Body mass index (BMI) was calculated by dividing weight in kilograms by height in meters squared. BMI was then categorized into underweight (BMI<18.5), normal weight (18.5 < BMI < 25.0), overweight (25<= BMI < 30.0) and obese (BMI>=30) (36). The height of each study participant was standardized by calculating their relative Z score, and consequently, patients with z score values below -2 were considered to have short stature. Ferritin level measured in μg/L was further classified into two groups using the threshold of 2500 μg/L (27). Endocrinopathies prevalence was compared between the two groups of Ferritin level using Chi-square test.

### Ethical approval

Data collection for this research commenced after granting ethical approval from Dubai Scientific Research Ethics Committee at Dubai Health Authority. The reference number for the approval letter is DSREC-06/2019-10.

## Results

### Characteristics of the cohort

A total of 200 adults with BTM between the ages of 19 and 35 years were enrolled in this study. The participants’ mean age was 27.5 years (SD = 4.5). Females constituted 54.5% (n=109) of the sample, while 45.5% (n=91) were males. The study sample included UAE nationals (36.0%, n=72), Arabs (27.2%, n=53), and non-Arabs (35.9%, n=70). 22.5% (n=45) of all participants were married with a mean marriage age of 25 years (SD = 3). Of all married adults, 60% (n=27) had children. More than half (59.0%, n=118) of the participants had normal weight status, 15.5% (n=31) were underweight, 15.1% (n=30) were overweight, and 10.0% (n=20) were obese. The mean height of all male patients was 165.4 cm (SD = 6.05), and for females it was 154.11 cm (SD = 5.67), while the mean weights of all male and female patients were 60.4 kg (SD = 12.74) and 55.8 kg (SD = 14.60), respectively. The mean weights and heights of the UAE male and female patients were compared to their respective mean values in the UAE adult population. For the UAE males (n=38), their mean height (164.5 cm, SD = 6.39) was significantly different from the mean height of the UAE adult male population (171 cm; t=-6.261, df=37, p-value<0.001) (37). Similarly, for the UAE female patients (n=39), their mean height (154.1 cm, SD = 5.35) was significantly lower than the average UAE female adult population (157.5 cm, t=-3.991, df=38, p-value<0.001). Moreover, the weight of the UAE male patients (60.1 kg, SD = 13.58) was significantly lower than the UAE male adult population mean weight (71.5 kg, t=-5.168, df=37, p-value<0.001). whereas the mean weight of the female UAE patients (56.8 kg (SD = 15.48) was comparable to that of the UAE female adult population (57.8 kg; t=-0.403, df=38, p-value=0.689) (37). (**Table 1**).

**Table 1:** Demographic and physical characteristics of study participants with beta thalassemia (N = 200).

	N	%
Age (years); Mean (SD)	27.5 (SD = 4.5)	
Sex		
Male	109	54.5%
Female	91	45.5%
Marital status		
Unmarried	139	69.5%
Married	45	22.5%
Not specified	16	8.0%
Had Children		
No	18	40.0%
Yes	27	60.0%
Nationality		
UAE	72	36.0%
Arabs	53	27.2%
Non- Arabs	70	35.9%
Not specified	5	2.5%
BMI groups		
Underweight	31	15.5%
Normal	118	59.0%
Overweight	30	15.1%
Obese	20	10.0%
Not specified	1	0.5%
Diagnosis		
BTM	188	94.0%
BTI	2	1.0%
HbH-AT	1	0.5%
SC-BT	5	2.5%
HbE-BT	2	1.0%
Not specified	2	1.0%
Age at Puberty (years); Mean (SD)	15.3 (SD = 2.7)	
Menses in females		
No	9	9.9
Yes	82	90.1
Height in UAE Males; Mean (SD)	164.5 cm (SD = 6.39)	
Height in UAE Females; Mean (SD)	154.1 cm (SD = 5.35)	
Weight in UAE Males; Mean (SD)	60.1 Kg (SD = 13.58)	
Weight in UAE Females; Mean (SD)	56.8 Kg (SD = 15.48)	

### Menarche & fertility in females

Of all female patients, the mean age of menarche was 15 years (SD = 4.0); 57.1% (n=52) had menarche at the age of 15 years or below while 42.9% (n=39) had menarche at an age above 15 years. Ferritin levels were comparable between females with normal and delayed menarche (median value of 3461 compared to 3328, respectively, U = 853, p-value = 0.775).

Among the 91 females, seven (7.7%) were using estrogen/progesterone supplements. These females exhibited a median puberty onset age of 17 years, with an interquartile range (IQR) of 3 years.

In this study, of the total of 91 females, nine (9.9%) did not have menses. Their age ranged between 19 and 34 with a mean of 28 years (SD = 5), their median ferritin level was 3634, two females were on DFO, one on DFP, and 7 on DFX chelation drug. Eight females were on a single chelation drug, while only one was on multiple drugs.

Fertility was assessed among married females. Among the females, 22% (n=20) were married, of whom 55% had children who were conceived using natural methods. Of the married women, 35% (n=7) had one child, 10% (n=2) had two children, and 10% (n=2) had three children.

### Pubertal onset & fertility in males

In male patients, the age of pubertal onset could not be ascertained as data on accurate testicular volume measurement were not available. The use of testosterone supplements was reported in 28.4% (n=31) of the males (n=109). Among these males, the median puberty age was 15 years (IQR = 3).

Married males represented 22.9% (n=25) of all males, and 64% of them had children. Of all fathers, only two children were conceived using the *In-Vitro* Fertilization (IVF) method, while the other children were born through natural methods.

### Clinical parameters

The average annual pre-transfusion hemoglobin level was low (<9 g/dL) in 18.0% (n=36) patients while the rest (81.5%, n= 163) had hemoglobin value of at least 9 g/dL. Short stature was reported for three cases only, two of whom were males and one was female (**Table 2**).

**Table 2:** Clinical characteristics of study participants with beta thalassemia (N = 200).

	N	%
Blood transfusion		
No	9	4.5%
Yes	191	95.5%
Chelation drugs		
DFO	47	23.5%
DFP	35	17.5%
DFX	155	77.5%
Number of chelation drugs		
0	7	3.5%
1	153	76.5%
2	36	18.0%
3	4	2.0%
Ferritin; Median (IQR)	2756 (3735)	
TSH		
< 0.3	1	0.5%
0.3 – 4.2	157	78.5%
> 4.2	37	18.5%
Not specified	5	2.5%
FT4		
< 12.0	5	2.5%
12.0 – 23.0	191	95.5%
> 23.0	4	2.0%
Hypothyroidism		
No	178	89.0%
Yes	22	11.0%
Hemoglobin		
< 9 gm%	36	18.0%
>= 9 gm %	163	81.5%
Not specified	1	0.5%
Diabetes Mellitus		
No	170	85.0%
Yes	30	15.0%
Hypogonadism		
No	154	77.0%
Yes	46	23.0%
Hypoparathyroidism		
No	182	91.0%
Yes	18	9.0%
Short stature		
No	197	98.5%
Yes	3	1.5%

Physical and clinical parameters were compared by sex. The prevalence of low hemoglobin (<9 g/dL) was comparable between males and females (20.2% and 15.6% in males and females, respectively, Chi-square = 0.713, p-value = 0.399). Yet, ferritin level was significantly higher in females (median = 3415) than in male patients (median=2163; U = 3700, p-value = 0.042).

Bivariate correlational analysis was conducted among the different clinical parameters. There were significantly negative, yet mild correlations between the level of Ferritin and each of Testosterone-Total, LVEF, and Transverse Relaxation time Magnetic Resonance Imaging (T2MRI)-Heart (spearman correlation coefficients, ρ, were -0.233, -0.203, and -0.328, respectively). Similarly, FSH and LH levels were significantly negatively correlated with age (ρ= - 0.228 and ρ= - 0.278, respectively) while T2MRI-Heart showed weak negative correlation with age at puberty (ρ= - 0.186, p-value=0.022) (**Table 3**). Yet, to avoid inflation of type 1 error resulting from multiple comparisons, after applying Bonferroni’s correction, the only correction that remained statistically significant was that between Ferritin and T2WI Heart (ρ= -0.328, p<0.001).

**Table 3:** Bivariate correlation analysis among clinical parameters of study participants.

	Spearman correlation coefficient	P-value
Ferritin level		
Height Z score	- 0.103	0.156
FSH	- 0.102	0.364
LH	- 0.117	0.297
Testosterone-Total	- 0.233	***0.015***
LVEF	- 0.203	***0.005***
T2WI Heart	- 0.328	<0.001**
FSH		
Age	- 0.228	***0.030***
Age at Puberty	- 0.091	0.416
Ferritin	- 0.102	0.364
LH		
LH		
Age	- 0.278	***0.008***
Age at Puberty	- 0.176	0.115
Testosterone-Total		
Age	0.019	0.848
Age at Puberty	- 0.090	0.393
LVEF		
Age	- 0.067	0.345
Ferritin	- 0.203	***0.005***
T2MRI Heart		
Age	- 0.005	0.944
Age at Puberty	- 0.186	***0.022***
TSH	- 0.031	0.692

Statistical significance was assessed using Bonferroni correction for multiple comparisons (α = 0.05/15 = 0.003).

**Significant p-value after Bonferroni’s correction.

Bold text indicates statistical significance.

**Figure 1:**
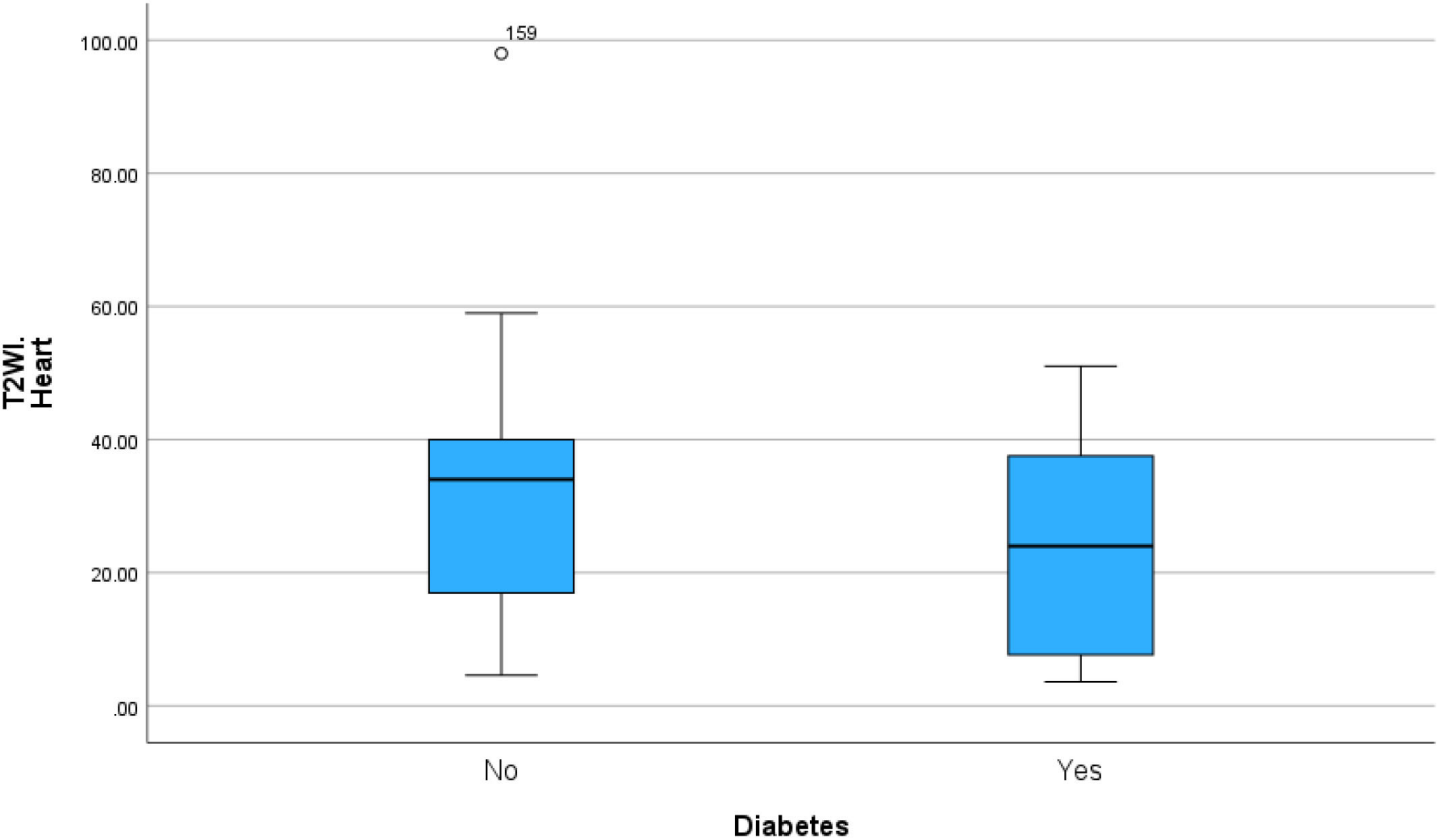
Boxplot showing the distribution of T2WI-Heart by Diabetes Status.

The median value of T2MRI-Heart was compared between patients with and without diabetes. The median T2MRI-Heart value was 34.00 among patients without diabetes, while it was 24.00 among those with diabetes (U = 1566.000, p-value=0.090) (**Figure 1**).

Clinical parameters were evaluated in females who had no menarche and were compared between females with and without menses. Among females with no menses, the median values of ferritin were 3634 (IQR = 4984), T2WI.Heart was 18.6 (IQR = 30.9), and LVEF was 60 (IQR = 6.3). However, comparing the ferritin level between females with and without menses did not show any statistical significance (3415 vs 3634, respectively, U = 232, p-value= 0.317). Similarly, FSH and LH values were compared between females with and without menses. Median values of FSH were 1.9 (IQR = 6) and 3.1 (IQR = 3.1) for females with and without menses, respectively (U = 845.500, p-value=0.955). Moreover, LH median values did not significantly differ by females’ menses status (median=1.5 for no menses, and 4.6 for menses group; U = 979.500, p-value=0.461). The prevalence of diabetes was 33.3% (n=3) in females with no menses compared to 18.3% (n=15) in those with menses (Chi-square = 1.156, df=1, p-value=0.373). Similarly, the prevalence of hypothyroidism, female hypogonadism, and hypoparathyroidism did not differ significantly by menses status in females.

### Disease management

Of all 200 study participants, 95.5% (n=191) were on blood transfusion. As for the chelation drugs, 23.5% (n=47) of the patients used Deferoxamine (DFO), 17.5% (n=35) used Deferiprone (DFP), and 77.5% (n=155) used Deferasirox (DFX) drug. The main chelation drug used was DFX (77.5%), which was used either as a single drug or in combination with other drugs. Among all the 200 participants, 76.5% (n=153) were on a single chelation drug, 18.0% (n=36) were on two drugs, 2.0% (n=4) were on three drugs, and 3.5% (n=7) were on the ferritin level median value was 2756 (IQR = 3735) (**Table 2**).

### Diabetes and thyroid profile

The prevalence of Diabetes Mellitus (DM) was 15.0% (n=30), Hypogonadism was 23.0% (n=46), and Hypoparathyroidism was 9.0% (n=18). Diabetes prevalence was 11.0% (n=12) among males and 19.8% (n=18) among females (Chi-square = 2.992, p-value = 0.084). Hypothyroidism and hypogonadism were comparable between male and female thalassemia patients, while hypoparathyroidism was significantly higher in females compared to male patients (14.3% and 4.6%, respectively, Chi-square=5.696, p-value=0.017) (**Table 4**). All endocrinopathies, including DM, Hypogonadism, Hypothyroidim, and Hypoparathyroidism were compared between the two groups of Ferritin level (below 2500 μg/L and ≥2500 μg/L), yet no significant differences in the prevalence of endocrinopathies between the two groups.

**Table 4:** Comparing physical and clinical parameters by sex among study participants.

	Males (N = 109)		Females (N = 91)		Test value	P-value
	n	%	n	%		
Age at Puberty; Median (IQR)	–		15.0 (4.0)			
Hemoglobin groups						
< 9 g/dL	22	20.2%	14	15.6%	0.713 *	0.399
>= 9 g/dL	87	79.8%	76	84.4%		
Ferritin; Median (IQR)	2163 (3565)		3415 (3818)		3700 ^&^	***0.042***
Diabetes Mellitus						
No	97	89.0%	73	80.2%	2.992*	0.084
Yes	12	11.0%	18	19.8%		
Hypothyroidism						
No	94	86.2%	84	92.3%	1.866*	0.172
Yes	15	13.8%	7	7.7%		
Hypogonadism						
No	82	75.2%	72	79.1%	0.424*	0.515
Yes	27	24.8%	19	20.9%		
Hypoparathyroidism						
No	104	95.4%	78	85.7%	5.696*	***0.017***
Yes	5	4.6%	13	14.3%		

*Chi-square test; ^&^Mann-Whitney U test.

Bold text indicates statistical significance.

Among female participants, FSH and LH had median values of 3.2 mIU/ml (IQR = 3.70) and 3.3 mIU/ml (IQR = 5.43), respectively, while among males, testosterone mean value was 5.01ng/ml (SD = 3.25). LVEF and T2WI-Heart median values were 62.5 (IQR = 7.00) and 33.6 (IQR = 24.40), respectively.

## Discussion

Iron overload related endocrine complications continue to impact the health of surviving BTM patients despite the introduction of oral iron chelation for more than two decades as evidenced in our study.

Although none of the participants in our cohort was short, among the UAE nationals, men were shorter and lighter and women were shorter as compared to the reference population suggesting a degree of growth retardation among those with BTM.

**Figure 2:**
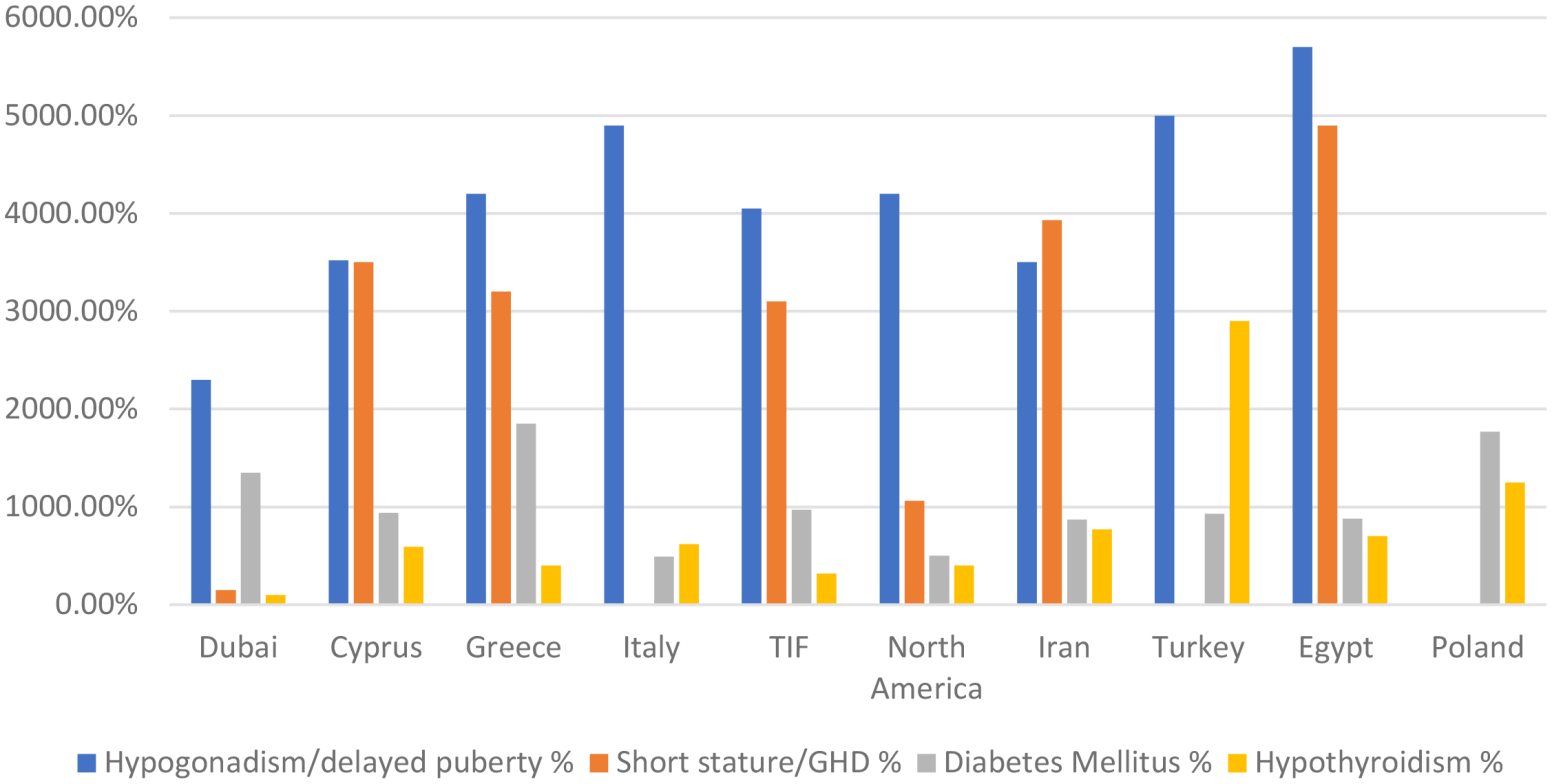
Endocrinopathies prevalence in different countries.

The prevalence of endocrinopathies is comparable to the international data (**Figure 2**) (38).

The mean age of menarche in UAE is 12.68 (SD 1.27) years twenty years ago, a more recent study found that the mean age at menarche was 11.5 years (± 1.17 SD) (39, 40). Despite the advancement in chelation therapy after the introduction of oral chelators in those with thalassemia major, females in our study had delayed menarche (mean age 15 years) compared to the normal population. In one study, 56 females with BTM had significant delay in menarche (14.5years) compared to control group (11.5years) (41).

Impaired glucose metabolism has been associated with higher ferritin levels (42). Recent studies have shown correlations between circulating insulin-like Growth factor 1 (IGF-1) and ALT levels and the risk of diabetes in this group of patients (43–45). The risk of diabetes among BTM patients ranges from 5.3 to7.7% worldwide but a higher prevalence in the Middle East reaching above 10% was reported (46). High serum ferritin particularly above 2500 µg/L seems like a risk factor in multiple studies (27, 47). Like other endocrine organs, pancreatic iron overload is toxic to the β-Langerhans cells (48). Cardiac iron overload can be one of the early predictors of diabetes development in these patients (49). While normal pancreatic T2 values have been associated with negative predictive values for glucose disturbance and with iron overload in the heart (50). This correlation seems to be higher in children and younger populations where the pancreas is relatively preserved. Higher body mass index has been associated with the risk of developing diabetes in patients with BTM (47, 51).

The impact of iron overload on the pituitary-gonadal axis and direct toxicity to the gonads has been established (52). Furthermore, high level of iron in the plasma negatively impact sperm motility (53).

The risk of complications from iron overload has decreased post the introduction of oral iron chelators namely Deferasirox and Deferiprone (54, 55). However, recent data from the region shows that almost one out of five young patients poorly complied with taking oral chelators (56).

On the other hand, good compliance with oral chelators has been shown to negatively correlate with serum ferritin level (57–60).

Hematopoietic stem Cell Transplantation (HSCT) remains the only curative treatment for BTM, however the decision to transplant a non-malignant condition like BTM especially with the availability of treatment (transfusion and chelation) is not a simple task because of multiple side effects on the short term such as acute graft versus host disease (GVHD) and rejection of HSCT or long term side effects such as chronic GVHD, major organ dysfunction and higher occurrence of solid tumors which is around six folds higher among patients who had transplant compared to non-transplanted patients (61). Hence, the focus should be on improving the compliance of current existing oral iron chelators and discovering potential new therapies targeting mechanisms of BTM at molecular level especially pertaining to mitochondrial pathways.

Improving compliance strategies can be implemented relatively sooner than new drug development. There are three main interventions’ strategies which are either behavioral interventions, educational interventions, or medications interventions (30). Behavioral modification around patients’ perceptions and attitude toward iron chelators, enhancing motivation to adhere to long-term therapy, advocating for adherence routines or reminders, encouraging family support, regular engagement of health care providers and shared decision making (60, 62). There are currently no data about educational intervention being evaluated to improve adherence to oral chelators. The only study showing that educational sessions significantly improve the quality of life of patients with BTM was conducted among patients receiving subcutaneous Deferoxamine (63). Strategies of medication intervention such as reducing or simplifying the treatment regimen whenever possible or alteration in chelators formulation has been shown to improve compliance. in one study, Deferasirox dispersible tablets were replaced by new film-coated preparation. This resulted in increase in adherence to therapy (92.9%) compared with 85.3% in the dispersible tablets’ group (64, 65).

Although there are no clinical trials examining the impact of targeting mitochondrial pathways in Thalassemia cases, there is surfacing data that may be utilized for potential new targeted therapies. Luspatercept; a drug released recently ameliorating anemia in transfusion dependent cases, gives a new hope for BTM patients. However the exact mechanism of action remains largely unclear (8, 66, 67). Animal studies are showing promising results with a drug called Mitapivat which is activate pyruvate kinase, hence reducing oxidative damage by increasing mitochondrial clearance (68, 69). Furthermore, there is promising data on animal models that drugs like Rapamycin and Sirolimus stimulates ULK1- autophagy pathway, subsequently increasing α- chain removal from cells (15, 16). Mitochondrial antioxidants namely Mitoquinone (MitoQ) is showing encouraging results in mice models by rescuing mitochondrial clearance through modifying ROS elimination (70). Currently, another drug approved by the food and drug administration (FDA) for cancer called Mitoxantrone is showing improvement in erythropoiesis in β-thalassemia mouse models (71). Another therapeutic potential though not yet explored in thalassemia may have a potential role, which is mitochondrial transplant to regain normal function (13, 72).

## Conclusion

The prevalence of endocrinopathies namely hypogonadism, growth failure, hypothyroidism, hypoparathyroidism, and diabetes mellitus still pose a major concern for patients with BTM despite the introduction of oral iron chelators. More emphasis needs to be placed on improving compliance with chelation, and further studies are required to investigate new therapies options to reduce these complications.

## Data Availability

The raw data supporting the conclusions of this article will be made available by the authors, without undue reservation.
